# “The usual suspects”- analysis of transcriptome sequences reveals deviating B gene activity in *C. vulgaris* bud bloomers

**DOI:** 10.1186/s12870-014-0407-z

**Published:** 2015-01-21

**Authors:** Anne Behrend, Thomas Borchert, Annette Hohe

**Affiliations:** Leibniz Institute of Vegetable and Ornamental Crops (IGZ), Department of Plant Propagation, Kuehnhaueser Strasse 101, 99090 Erfurt, Germany; Present address: Siemens Healthcare Diagnostics Holding GmbH, Ludwig-Erhard-Straße 12, 65760 Eschborn, Germany

**Keywords:** 454 sequencing, Bud flowering, Floral organ identity, Heather, Homeotic mutant, Real-time PCR, Transcriptome, Transcription factor

## Abstract

**Background:**

The production of heather (*Calluna vulgaris*) in Germany is highly dependent on cultivars with mutated flower morphology, the so-called diplocalyx bud bloomers. So far, this unique flower type of *C. vulgaris* has not been reported in any other plant species. The flowers are characterised by an extremely extended flower attractiveness, since the flower buds remain closed throughout the complete flowering season. The flowers of *C. vulgaris* bud bloomers are male sterile, because the stamens are absent. Furthermore, petals are converted into sepals. Therefore the diplocalyx bud bloomer flowers consist of two whorls of sepals directly followed by the gynoecium.

**Results:**

A broad comparison was undertaken to identify genes differentially expressed in the bud flowering phenotype and in the wild type of *C. vulgaris*. Transcriptome sequence reads were generated using 454 sequencing of two flower type specific cDNA libraries. In total, 360,000 sequence reads were obtained, assembled to 12,200 contigs, functionally mapped, and annotated. Transcript abundances were compared and 365 differentially expressed genes detected. Among these differentially expressed genes, *Calluna vulgaris PISTILLATA* (*CvPI*) which is the orthologue of the *Arabidopsis* B gene *PISTILLATA (PI)* was considered as the most promising candidate gene. Quantitative Reverse Transcription Polymerase Chain Reaction (qRT PCR) was performed to analyse the gene expression levels of two *C. vulgaris* B genes *CvPI* and *Calluna vulgaris APETALA 3* (*CvAP3*) in both flower types. *CvAP3* which is the orthologue of the Arabidopsis B gene *APETALA 3* (*AP3*) turned out to be ectopically expressed in sepals of wild type and bud bloomer flowers. *CvPI* expression was proven to be reduced in the bud blooming flowers.

**Conclusions:**

Differential expression patterns of the B-class genes *CvAP3* and *CvPI* were identified to cause the characteristic morphology of *C. vulgaris* flowers leading to the following hypotheses: ectopic expression of *CvAP3* is a convincing explanation for the formation of a completely petaloid perianth in both flower types. In *C. vulgaris, CvPI* is essential for determination of petal and stamen identity. The characteristic transition of petals into sepals potentially depends on the observed deficiency of *CvPI* and *CvAP3* expression in bud blooming flowers.

**Electronic supplementary material:**

The online version of this article (doi:10.1186/s12870-014-0407-z) contains supplementary material, which is available to authorized users.

## Background

*Calluna vulgaris* (Ericaceae) is an important ornamental crop for autumn planting in Northern Europe. The demand for *C. vulgaris* has constantly been increasing during the last years because of the longevity of a special mutant in flower morphology, the so-called bud bloomers. Today, 80% of all protected varieties of *C. vulgaris* in Germany are bud bloomers [1] and make *C. vulgaris* one of the top selling landscaping plants in Germany [2]. The bud bloomers show an unique flower architecture with combination of unopened flowers and absence of any organ development in whorl III: the perianth of bud bloomers remains closed during the whole flowering period, stamens are missing and petals are converted into sepals [3]. Bud blooming individuals were found in natural populations in 1936 and 1948 in Great Britain as well as in 1970 in the Netherlands [4] and were introduced as commercial varieties. Due to the shielding from cross-pollination by closed perianth organs and the impossibility of self-pollination due to the loss of stamens and the presence of a second whorl of robust sepals instead of softer petals, the flower buds of bud bloomers display a prolonged flower attractiveness compared to other flower types of *C. vulgaris*. The extended longevity of flowers is a highly desired trait promoting the bud bloomers’ economic success compared to varieties with wild type or filled flowers. An attractive flower morphology is one of the major selection targets in ornamental breeding.

**Figure 1:**
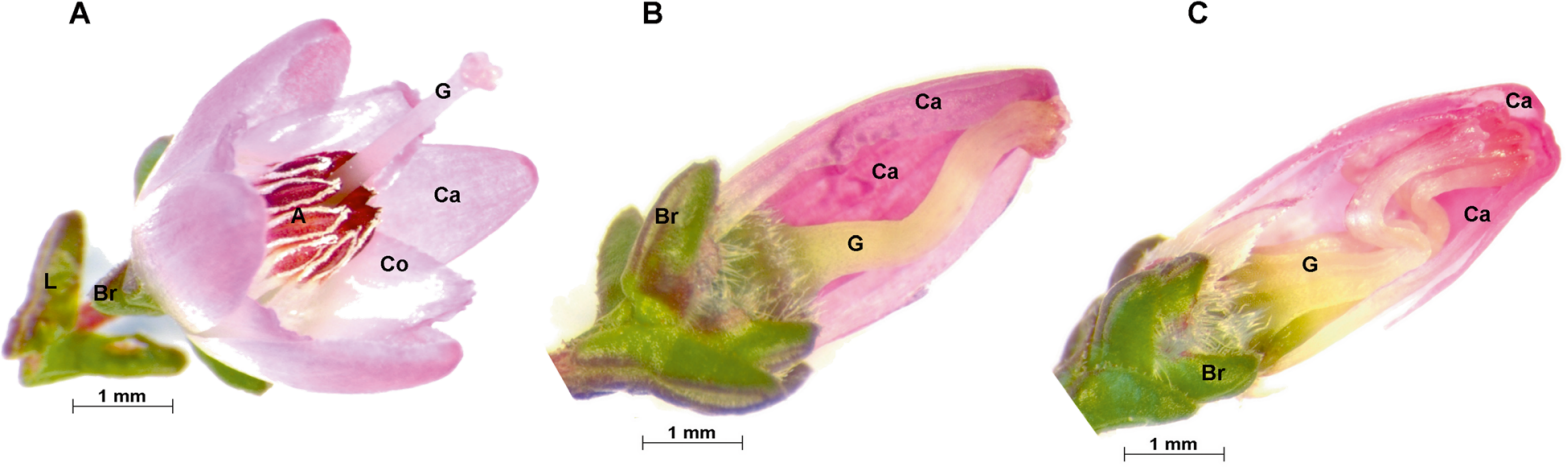
***C. vulgaris*** **flower types.** Flowers of *C. vulgaris*, **A** - wild type flower with leaves (L), flower organs to the centre: bracts (Br), sepals (Ca: calyx), petals (Co: corolla), stamens (A: androecium), and carpels (G: gynoecium), **B** - bud bloomer’s flower, diplocalyx type, flower organs to the centre: bracts (Br), sepals (Ca), sepals (Ca), and carpels (G), **C** - bud bloomer’s flower, polystyla type, cultivar ‘David Eason’, flower organs to the centre: bracts (Br), sepals (Ca), sepals (Ca), carpeloid stamens (G), carpels (G).

Within the bud bloomers two different types are found: the diplocalyx type and polystyla type [1,5] (Figure 1B and C). The diplocalyx type is by far dominating the market. The inheritance of the bud flowering diplocalyx type was found to be monogenic-recessive [6]. It is characterized by a closed perianth during the whole flowering period, stamens are completely missing and petals are converted to sepals. Hence, in the diplocalyx bud bloomer type, the two whorls of sepals are directly following the gynoecium. In floral development of this flower type, stamen primordia are detectable but stamens are not formed at all [3]. The floral formula of this type is Ca4+4Co0A0G(4) (Figure 1B, Ca: calyx; Co: corolla; A: androecium; G; gynoecium) [3], whereas the flower formula of the mature wild type (Figure 1A) is Ca4Co(4)A8G(4) [3]. In the polystyla bud blooming flower type the perianth remains closed and petals are converted to sepals as in the diplocalyx type, but organs in floral whorl III are formed and show carpel character (Figure 1C). The according floral formula is Ca4+4Co0G8G(4).

The genetics of different flower architectures can be explained by the ABC model of floral organ identity and its variants. It describes the interaction of the homeotic transcription factors in determination of floral organ identity [7-9]. In the classical ABC model, the expression of A genes is responsible for development of sepals in whorl I, activity of B genes in combination with C genes is necessary to determine organ identity of stamens in whorl III. B gene together with A gene function induces the formation of petals in whorl II. Finally, C gene expression on its own defines carpels. Since B genes in combination with A und C are responsible for the determination of organs in whorl II and III, and these organs are affected in both bud bloomer mutants, a deficiency in B gene function is the most convincing hypothesis for formation of the bud flowering phenotype in *C. vulgaris*. Accordingly, the polystyla bud blooming type corresponds perfectly to the phenotype of a classical B gene mutant as described in *Arabidopsis thaliana* (thale cress) [10], *Antirrhinum majus* (snapdragon) [11,12], and several other plant species [13-25]*.* The closed perianth in bud bloomers is probably the result of petal loss, as studies in *Arabidopsis* B gene mutants show [26].

In first gene expression analyses, Borchert et al. (2009) [3] already found a reduced expression of the B gene *CvAP3* in floral organs of whorl II in three diplocalyx bud flowering cultivars indicating the presence of a second whorl of sepals instead of petals which is expected according to the model. On the other hand, the formation of petaloid sepals in all flower types of *C. vulgaris* points to an ectopic expression of B genes in whorl I, resulting in conflicting hypotheses with regard to the genetics of the diplocalyx bud flower type.

Therefore, the aim of the current study was to compare the transcriptome of the wild type (wt) and the diplocalyx bud bloomer flowers (bud) of *C. vulgaris* and to deduce a hypothesis for the genetic basis of the diplocalyx bud bloomer flower architecture.

## Results

### 454 sequencing and assembly

**Figure 2:**
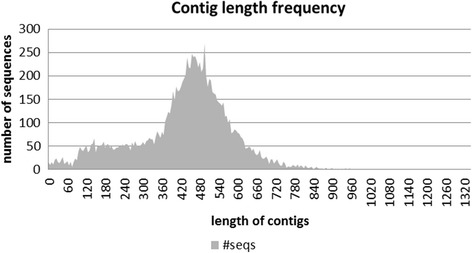
**Contig length.** Distribution of contig lengths after assembly of all 454 sequences reads.

For transcriptome comparison, the bud blooming cultivar ‘Maria’ (bud) and its wild type flowering descendent F1 (wt), resulting from a cross between ‘Maria’ and ‘Boskoop’ , have been selected in order to keep the genetic difference not depending on the flower type as low as possible. Two cDNA libraries were constructed from mRNA of young flower buds of both genotypes. Flowers included bracts, sepals, petals, stamens (from wt only), and carpels. The generated cDNA had a size of approximately 500–650 base pairs (bp). Libraries were tagged, combined and sequenced using the 454 sequencing technique (vertis Biotechnologie AG, Freising). A summary of sequencing and assembly results is given in Table 1. Overall, a total of 357,663 reads were generated with a total yield of ~ 110 Million nucleotides (Mnt). The average read length was 307 nt. Sequences shorter than 50 nt were not used in the assembly. The assembly of all reads resulted in 12,238 contigs (Table 1). Contig length was Gaussian distributed with a clear maximum around 500 nt (Figure 2). The separate assembly of the bud bloomer library resulted in 7,504 contigs, whereas the wild type library yielded 6,561 contigs after read assembly (Table 1). Singletons were excluded from further analysis since singletons are single reads without any significant overlaps with any other read. Therefore, it was considered as dubious to conclude differential gene expression from a single read. 4,352 common contigs were found in the wt library and the bud library.

**Table 1:** **Overview on 454 data**

**Assembly**	**Backbone**	**wt**	**Bud**
Assembled reads	278734	107013	145698
Total read number in contigs	246775	77220	118309
Number contigs	12238	6561	7504
Average length contigs (nt)	429	425	432
Number isotigs	11128	6070	6984
Average length isotigs (nt)	599	477	482
Number singeltons	30310	28991	26586
Average length singeltons (nt)	308	309	308

### Annotation of sequences

For annotation, contig sequences were compared to known sequences in publicly available databases. Blast2go [27] was used for blasting, mapping, and annotating the contigs by comparing the assembled sequences to the non-redundant protein (nr) data base of the National Centre for Biotechnology Information (NCBI) (Table 2). From the assembly of all reads (backbone), 63.8% of the contigs shared significant homology with known proteins. 67.7% of the contigs from the bud library and 67.6% of the contigs from the wt library, respectively, showed significant homology to proteins from the database. In all assemblies, around 5.6% of the contigs displayed homology to unknown/hypothetical proteins. Most BLAST hits were obtained from *Vitis vinifera* (grape), followed by *Glycine max* (soybean), *Populus trichocarpa* (black cotton wood), *Arabidopsis thaliana* (thale cress), and *Cucumis sativus* (cucumber). *Vitis vinifera* is the closest phylogenetic relative of *C. vulgaris* with a completely sequenced genome available. The amount of BLAST hits is correlated to the amount of available sequence information. Therefore, closer relatives of *C. vulgaris* like *Camellia sinensis* (tea), *Actinidia chinensis* (yellow kiwi fruit), or *Actinidia deliciosa* (green kiwi fruit) delivered BLAST Top-Hits (Additional file 1), but were outnumbered by fully sequenced organisms.

**Table 2:** **Number of contigs in each library during processing in blast2go**

**Status**	**Backbone**	**wt**	**Bud**
Without BLAST hit	4431	2125	2426
With BLAST result	667	356	388
With mapping result	864	448	492
Annotated sequences	6276	3632	4198
Total number	12238	6561	7504

### Differential gene expression

**Figure 3:**
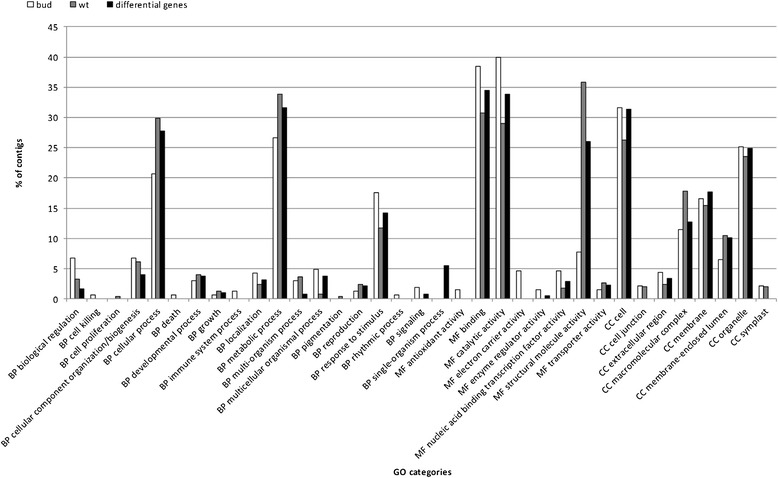
**Functional annotations based on GO categories of contigs from wt library, bud library assembly, and contigs differently expressed in** ***C. vulgaris*** **bud bloomer and wild type flowers.** BP – biological process, MF – molecular function, CC – cellular component.

**Figure 4:**
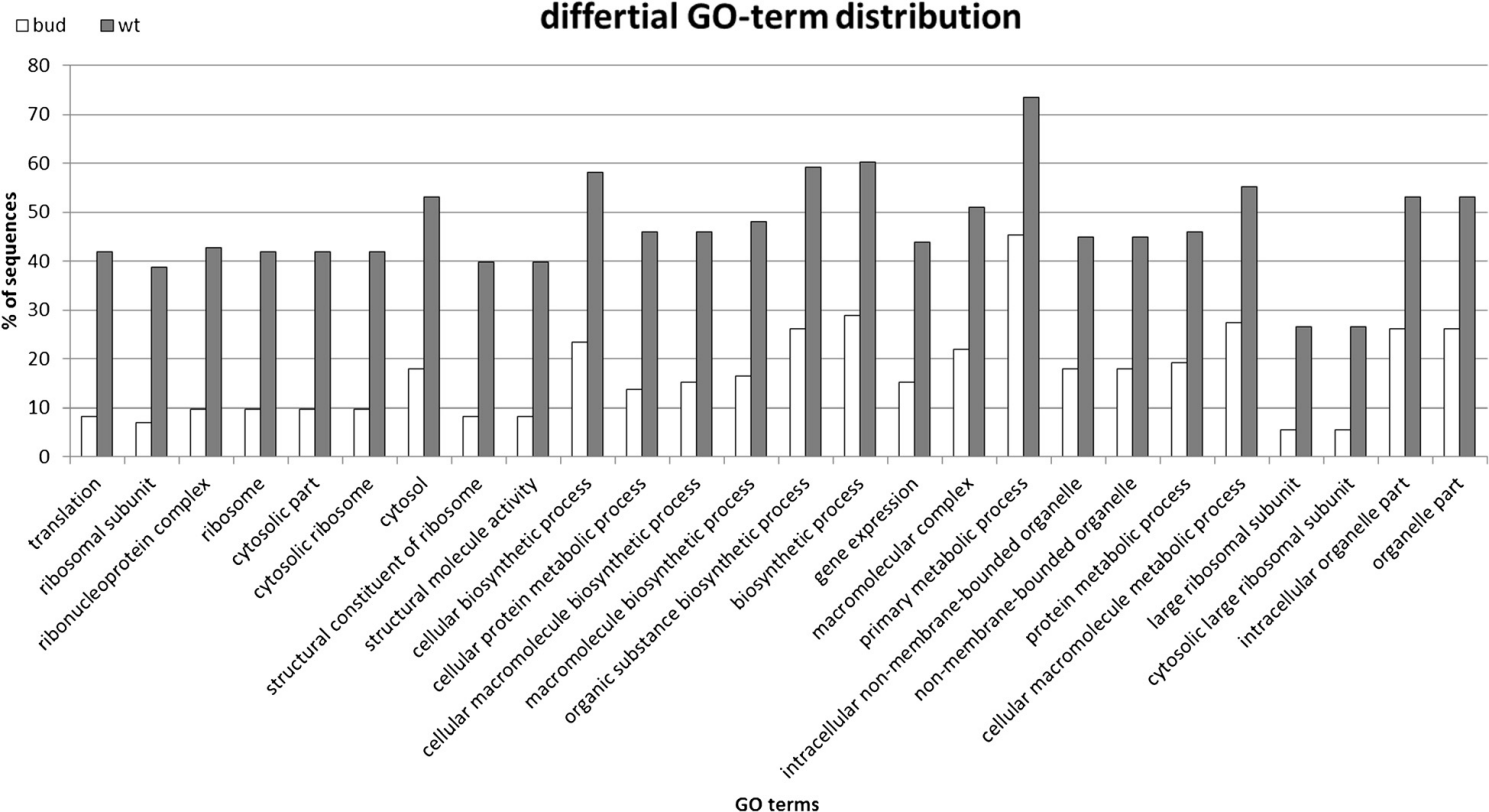
**Differential GO term distribution among differentially expressed genes.** GO term enrichment analysis by Fischer’s exact text.

The primary goal of the transcriptome study was to identify differentially expressed genes in both flower types and to obtain sequence information of *C. vulgaris* for later identification and validation of possible candidate genes. Flower type specific read numbers per contig were obtained by mapping the flower type library reads to the backbone assembly. Subsequently the transcript abundances in both libraries were compared. To discover genes uniquely or preferentially expressed in one of the flower type specific libraries, Audic Claverie statistics [28] via the web tool IDEG6 [29] was used. 365 contigs were found to be statistically significant differentially expressed comparing the bud flowering and the wild type phenotype (Additional file 2). 178 contigs were found to be preferentially expressed in the bud bloomer and 88 of these were found exclusively in the bud flowering phenotype. In the wild type, 187 contigs were preferentially expressed and 50 were found to be present only in this flower type. Sequences with significant similarities to annotated proteins in NCBI were assigned to the Gene Ontology (GO) categories biological process, molecular function, and cellular component (Figure 3). Homologues proteins involved in biological processes were attributed to metabolic processes, cellular processes, responses to stimulus, biological regulations, and cellular components organisation or biogenesis. Regarding the molecular functions, catalytic activities and binding properties were the most abundant GO categories followed by transporter activities, structural molecular activities, and electron carrier activities. With respect to the cellular components, homologues proteins were mostly associated to organelles, membranes and macromolecule complexes. For more detailed analysis, a GO enrichment analysis by Fischer’s exact test was performed and revealed for differentially expressed genes in the wt data set an overrepresentation of the GO terms translation, ribosomal subunit, ribonucleoprotein complex, ribosome cytosolic part, cytosolic ribosome, cytosol, structural constituent of ribosome, structural molecule activity, cellular biosynthetic process, and cellular protein metabolic process (Figure 4).

### Functional classification of differentially expressed genes

171 differentially expressed genes (46.8%) did not match homologues proteins in the data base. Differentially expressed genes that could be annotated were checked for functional classification in biological processes to identify reasonable candidates for the bud flowering phenotype. GO enrichment analysis pointed out to overrepresentation of GO terms related to ribosome function in wt. In addition, the data sets of differentially expressed genes in the GO categories “flower development”, “floral whorls development” and “sequence specific DNA binding transcription factor activity” were carefully checked for probable candidate genes (Additional file 3). The following annotated contigs were assigned to flower or floral whorl development: *DNAj, glycerol-3-phosphate acyltransferase, 26S proteasome non ATPase regulatory subunit rpu 12a, basic blue protein, 3-ketoacyl-synthase 6*. None of these was considered as potential candidate gene for the bud flowering phenotype. In addition, the BLAST and mapping results of four differentially expressed transcription factors were monitored. Two putative transcription factors, a GAGA binding transcriptional activator and an ethylene responsive transcription factor RAP2-3, are considered to be involved in stress response. A putative E2FE like transcription factor is involved in cell proliferation. Consequently, these three genes were also excluded as candidate genes. The fourth one, contig07420 which exerts a homology to *PISTILLATA (PI),* belonging to the class B genes*,* of *Actinidia chinensis* (yellow kiwi fruit), was identified as a promising candidate gene and was named *CvPI*.

The genes differentially expressed in the different flower type of *C. vulgaris* were also compared to a list of differentially expressed genes in *Arabidopsis* B gene mutants from microarray studies [30]. 51 of the contigs from *C. vulgaris* could be assigned to counterparts in the *Arabidopsis* data set, at least on protein family level. Most matches (20) were obtained with the *pi-1* mutant. 16 matches were found with *ap3-1* mutant and 15 with the *ap3-3* mutant. 45 *C. vulgaris* contigs showed a similar expression pattern as the corresponding genes in *Arabidopsis* in at least at one of three time points monitored in the *Arabidopsis* study (Additional file 2).

### Evaluation of candidate gene by real-time PCR (qRT PCR)

**Figure 5:**
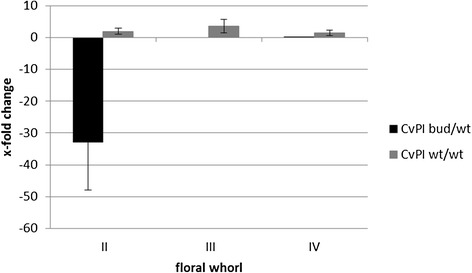
**Expression pattern of** ***CvPI*** **.** Normalised gene expression (five reference genes) in the bud blooming phenotype and the wild type shown as fold change (2^-ΔΔCt^) of arbitrary units compared the reference tissue of F1 (wt).

**Figure 6:**
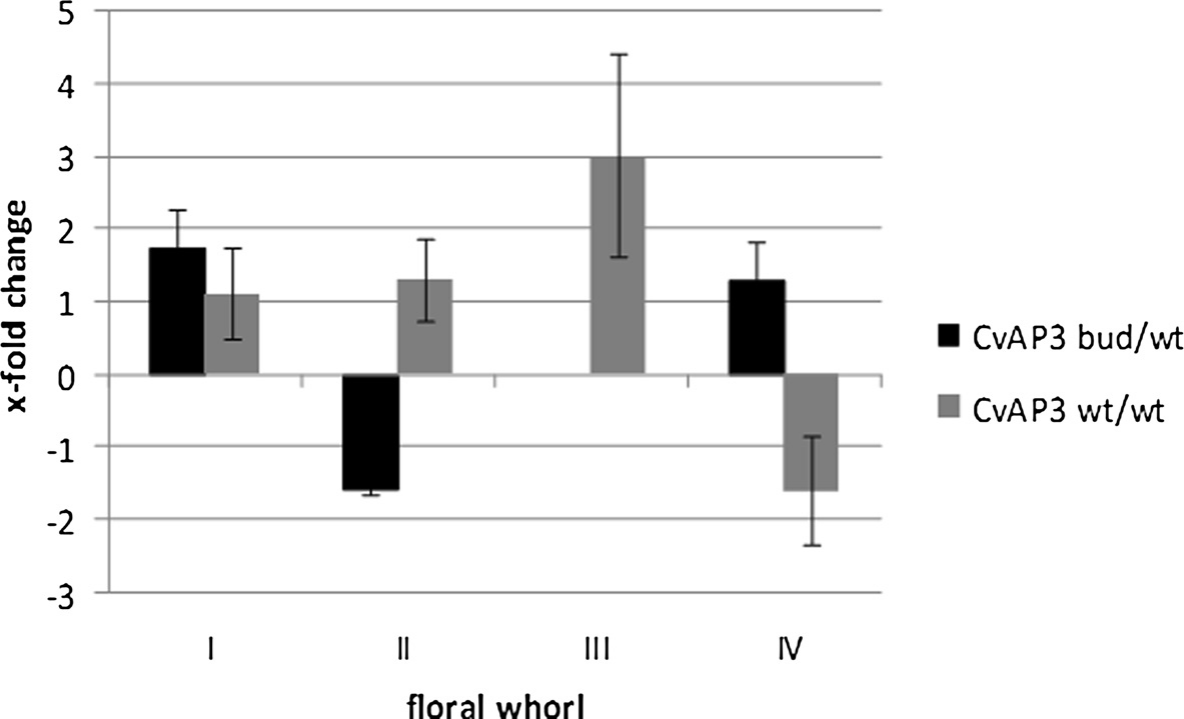
**Expression pattern of** ***CvAP3.*** Normalised gene expression (five reference genes) in the bud blooming phenotype and the wild type shown as fold change (2^-ΔΔCt^) of arbitrary units compared the reference tissue of F1 (wt).

For subsequent validation of *CvPI* function in *C. vulgaris* flower organ formation, a quantitative PCR analysis was performed in wild type and three bud blooming genotypes. Although transcriptome data gave no hint on differential expression in the bud and wt libraries for the second identified B gene from *C. vulgaris*, *CvAP3* [3] was also included in the study, as Borchert et al. 2009 [3] found deviating expressions patterns of *CvAP3* in floral tissues of diplocalyx *C. vulgaris* bud bloomers and wild type cultivars. Five reference genes were chosen from the library. The reference genes with most stable expression in flower tissue were: *CvTATA binding, Cv18S rRNA*, *CvActin*, *CvTSa*, and *Cvdisease resistance protein*. To compare flower type specific expression of *CvPI* and *CvAP3* ΔΔCt values were calculated with F1 (wt) as reference and converted to fold change ratios of arbitrary units. Exemplarily for the three different cultivars per flower type, the results of the cultivars ‘Maria’ (bud, pistillate parent) compared to the genotype F1 (wt, offspring) and ‘Boskoop’ (wt, staminate parent) compared to genotype F1 (wt, offspring) are presented (Figures 5 and 6). These genotypes were chosen, because the cDNA libraries for transcriptome sequencing were generated from ‘Maria’ (bud) and F1 (wt). *CvPI* expression in both phenotypes was no accurately detectable in leaves, bracts and sepals, whereas *CvAP3* expression was found in all studied organs. Hence, differential gene expression data of *CvAP3* for all floral whorls are presented in Figure 6, whereas corresponding data of the sepals (whorl I) for *CvPI* in Figure 5 are missing. *CvPI* showed the expression pattern expected from the ABC model with the highest expression level in whorls III and II of wild type flowers, thus confirming the results of the transcriptome analysis, since the expression of *CvPI* was reduced in floral organs of bud bloomer ‘Maria’. Although the expression level of *CvPI* was found to be genotype dependent, a clear organ specific expression pattern was identified in all genotypes. Reliable expression data of *CvPI* were obtained from wt flowers in whorl IV, whorl III, and whorl II organs; in bud bloomers in whorl IV and whorl II. In wt flowers of F1 and ‘Boskoop’ , *CvPI* expression was most abundant in whorl III followed by whorl II and whorl IV. Compared to the expression of *CvPI* in wt flowers in whorl II, its expression in the diplocalyx bud bloomer was reduced by factor 32 (Figure 5). Likewise, in the transcriptome analysis 20 sequences reads of *CvPI* were obtained from the wt library (F1) and none in the bud bloomer’s library (‘Maria’). This reduction of *CvPI* expression was not observed comparing F1 and ‘Boskoop’ (Figure 5). In contrast, the expression of *CvAP3* was clearly detectable throughout all floral organs in both flower types (Figure 6). Fold-change ratios comparing expression of *CvAP3* in the different flower types were generally smaller than the corresponding values for *CvPI*. As expression of *CvAP3* on whole flower level did not clearly differ between the flower types, no differential expression was detected in the transcriptome approach. In the organ-specific qRT PCR analysis, the bud bloomer showed a lack of *CvAP3* expression in whorl II compared to the wt F1, so both, *CvAP3* and *CvPI* expression are reduced in whorl II of bud bloomers. The comparison of the male parent ‘Boskoop’ and its offspring F1 indicates a lower abundance of *CvAP3* expression in organs of whorl I-III but a higher expression in whorl IV. In ‘Maria’, the bud blooming parent of F1, *CvAP3* expression compared to its wt offspring was higher in whorl I and IV but reduced in whorl II and not detected in whorl III, since the organs are absent. The overall highest fold-change ratio for differential expression of *CvAP3* was factor 3, detected in stamens of different wild type genotypes, indicating that the detected fold-change ratios of *CvAP3* cannot be clearly attributed to differences of the flower types or genotypic differences independent of the flower type. Expression of *CvAP3* in whorl I seems to be common for *C. vulgaris*, since wild type and bud bloomer exhibit *CvAP3* transcript levels in a comparable abundance in whorl I (sepals). The reduction of *CvAP3* expression in whorl II of bud bloomers confirms earlier findings demonstrating that bud bloomer organs in whorl I and II are sepals [3] instead of sepals and petals in the wild type. In both flower types, expression of *CvAP3* is deviating from the classical ABC model showing ectopic B gene expression in whorl I.

## Discussion

In *C. vulgaris* bud bloomers of the diplocalyx type male flower organs are missing (Figure 1B), petals are converted into a second whorl of sepals and the flower remains closed. This flower type is a highly desired trait in ornamental plant breeding, since the bud bloomers’ flowers have an extended flowering period. The aims of this study were to characterise the gene expression profile of *C. vulgaris* diplocalyx bud bloomers by a broad transcriptome study and deduce candidate genes causing the diplocalyx bud flowering phenotype by the comparison with wt flowers.

Unopened flowers which later drop and form no siliques have been described in Arabidopsis *LSU4* mutants [31]. In addition, flowers of *pi-1* mutants as well as transgenic *Arabidopsis* plants ectopically expressing *LMADS8* or *LMADS9* flowers were termed as unopened [32]. The morphology of these mutants points to a crosslink of floral organ morphology and flower opening [26,33]. This circumstance is a good explanation for the bud bloomers’ phenotype in *C. vulgaris*. Since stamen development was not detectable in the diplocalyx type [3] or stamens have carpel-like character in the polystyla type and petals are replaced by sepals, organs responsible for flower opening are missing in these flower types. The identity of the affected organs points to a modified expression of a B gene in the bud flowering phenotype, since stamens and petals are the mutated organs. The apparent absence of third whorl organs may reflect their complete incorporation into the fourth whorl gynoecium [34]. Upstream regulators of B gene expression as *UFO*, *LEAFY* or *AP1* are unlikely to be affected in the *C. vulgaris* bud bloomer mutants, because dysfunctions in these genes would cause severe flower malformations: *UFO* mutants in *Arabidopsis* display filamentous structures instead of flowers [35]. *LEAFY* mutants produce leafs and associated lateral shoots instead of early flowers, later developing flowers are substituted by structures with flower and leaf traits [36,37]. In *AP1* mutants of *Arabidopsis*, sepals are replaced by bracts, petals are missing and additional flowers arise in the axils of the first whorl organs [38,39].

However, in model plants, typical B gene loss of function mutants display a second whorl of sepals instead of petals and the formation of carpeloid stamens. In *Arabidopsis*, the B genes *APETALA3* (*AP3*) and *PISTILLATA* (*PI*) are responsible for the control of organ identity in whorl II (petals) and III (stamens) [10]. Since *AP3* and *PI* function as a heterodimer in *Arabidopsis*, mutations of either *AP3* or *PI* cause identical phenotypes with altered organ identity in whorl II and whorl III [10]. The function of the B class genes *AP3* and *PI* seems to be highly conserved during evolution of flowering plants. Because *C. vulgaris* bud bloomers phenotype shows conflicting characters compared to a classical B gene mutant - on the one hand petaliod sepals, on the other hand loss of stamens and petals - a broad RNA sequencing approach was chosen to find genes differentially expressed in wt and the diplocalyx bud flowering phenotypes of *C. vulgaris*. These data have been compared to the data set of Wuest et al. 2012 [30] to elucidate parallels and differences with *Arabidopsis* B gene mutants.

High throughput 454 sequencing was found to be an effective method to characterise the transcriptomes of different flower types of *C. vulgaris*. Next generation sequencing is the state of the art approach for broad gene expression analysis relative to methods such as microarrays and subtractive cDNA libraries [40-42]. The 454 sequencing technology is an effective tool for tissue specific functional genomics in non-sequenced plants species, because it is capable to capture also rarely expressed transcripts as transcription factors [43-49] and delivers massive numbers of additional transcript sequences which were useful in the presented study for qRT PCR normalizer choice. In addition, the obtained data bases of *C. vulgaris* floral transcriptomes are valuable resources for further research on flower related traits in this ornamental crop.

From the set of 365 differentially expressed genes, *CvPI* was considered to be the most plausible candidate responsible for causing the diplocalyx flower mutant. Moreover, a significantly reduced expression level of *CvPI* in diplocalyx bud bloomers has been confirmed by qRT-PCR. Nevertheless, the lack of *CvPI* expression in *C. vulgaris* bud bloomers is not causing the typical phenotype of a B gene mutant as anticipated from *Arabidopsis*, since in diploxcalys flower mutants, stamens are completely missing. A similar phenotype has been found in a peloric mutant of *Phalaenopsis equestris* in which the development of stamens and staminodes was completely eliminated [50] and the expression of the B gene *PeMADS5* was not detectable in the floral tissue.

In *C. vulgaris,* the expression of *CvPI* was found to be high in petals and stamens of the wild type as expected from the ABC model. In contrast, *CvAP3* expression was prominent in whorl I-III of wt and diplocalyx bud blooming flowers. In opposite to *CvAP3*, hardly any *CvPI* transcript was detectable in the floral tissues of the bud flowering plants by qRT PCR. According to the classical ABC model and its modifications, the expression of the *AP3*-like gene is restricted to whorls II and III [7]. *CvAP3* expression in whorl I is considered to cause the petaloid character of *C. vulgaris* sepals in both studied flower types. This finding is supported by earlier expression analysis in *C. vulgaris* [3] and data from multiple species, including important floriculture crops as *Tulipa gesneriana* (garden tulip) [51], *Lilium longiflorum* (Easter lily) [52], and *Agapanthus praecox* (common agapanthus) [53]. Since *CvPI* expression is absent from floral tissue of diplocalyx bud bloomers, it is assumed that petal and stamen development in *C. vulgaris* depends on the binding of *CvPI* in a regulatory complex of MADS box genes containing *CvAP3* and the absence of *CvPI* is causing to the development of a second whorl of petaloid sepals and the absence of stamens*.* Due to the petaloid character of this extra whorl of sepals and the expression level of *CvAP3* in whorls II and III, it is concluded that only the lack of *CvPI* expression is causing the altered flower architecture and not a combined dysfunction of *CvPI* and *CvAP3*. In addition, the finding of *CvAP3* transcripts in carpels of *diplocalyx* bud bloomers without stamen character also points to the hypothesis of an exclusive dysfunction of *CvPI* being responsible for the loss of stamens.

To elucidate the consequences of putative *CvPI* dysfunction in *C. vulgaris* the list of differentially expressed genes in young flowers of *C. vulgaris* comparing the diplocalyx bud bloomer and wild type flowers was compared to published data from *Arabidopsis* B gene mutants [30]. In this study 2100 genes were identified to be differentially regulated in B gene mutants. In *Arabidopsis pi1-1* mutants, GO terms like petal development, stamen development, floral organ formation, floral organ morphogenesis, and regulation transcription were found to be significantly enriched. In contrast, these GO terms were not enriched in the *C. vulgaris* data set. The major difficulty in functional analysis of differentially expressed genes in *C. vulgaris* bud bloomers proved to be the low informative value of GO term enrichment analysis. The annotation of *C. vulgaris* sequences did not identify single genes but gene families or only protein motives, making obtained GO terms rather unspecific. This is attributed to the low sequence identity between *C. vulgaris* and model plants and to the incomplete annotation of sequence data from closer relatives. Therefore, for more detailed results using GO analysis of the present 454 read data, more detailed sequence information of *C. vulgaris* or close relatives is needed.

Further studies on the bud bloomers phenotype in *C. vulgaris* are planned including comparison of B gene expression in the diplocalyx and polystyla type and the localisation of transcripts with an in situ hybridisation approach to unveil *CvPI* and *CvAP3* expression pattern during floral development. Protein and DNA binding studies with *CvPI* and *CvAP3* protein from bud bloomer and wild type genotypes are necessary to clarify the composition and function of homeotic floral MADS box protein complexes *in C. vulgaris* flower development. Of special interest in *C. vulgaris* is the investigation of the crosslink of B gene expression and the genetic regulation of carpel development reported from *Arabidopsis* [30], since several cultivars with bud flowering phenotype suffer from carpel malformation [54]. Moreover, mapping of *CvPI* expression in an existing mapping population [55] is planned to check the cosegregation with the trait flower type.

## Conclusions

The B genes *CvPI* und *CvAP3* have been found to play crucial roles in the development to the diplocalyx bud bloomer mutants of *C. vulgaris*, which are of major economic significance in this important landscaping plant. Ectopic expression of *CvAP3* in sepals seems to be responsible for their petaloid character. A drastically reduced expression of *CvPI* in flowers of diplocalyx bud bloomer mutants points to a central role of this transcription factor in the formation of this flower type. Further research is necessary to figure out the differences in B gene expression between polystyla and diplocalyx bud bloomers in *C. vulgaris*.

## Methods

### Plant material

Plants of bud flowering varieties (‘Maria’ , ‘Anett’ , ‘Marlis’ , ‘Ginkel’s Glorie’) and genotypes with wild type flowers (‘Boskoop’ , ‘Hammonidii’ , F1, Niederohe) were kept in the IGZ greenhouse in winter and under field conditions in frost free periods. ‘Maria’ , ‘Anett’ , ‘Marlis’ , ‘Ginkel’s Glorie’ , Boskoop’ , ‘Hammonidii’ are commercially available varieties. The wild type Niederohe was grown from plant material collected in Germany. The genotype F1 originated from the cross ‘Maria’ x ’Boskoop’. Flowers from all genotypes were collected and dissected into bracts, sepals, petals, stamens (if present) and carpels. Floral organs and leaves were conserved in RNAlater (Invitrogen) and stored at −80°C.

### RNA extraction and cDNA synthesis

Total RNA was extracted with the RNeasy Plant Mini Kit (Qiagen) according to the manufacturer’s instructions with modifications as published in Dhanaraj et al. (2004) [56] including intensively on column washing with 80% EtOH. The complete digestion of genomic DNA was performed using TurboDNase (Ambion) according to the manufacturer’s protocol. RNA was quantified using the Nanodrop spectrometer (Thermo Scientific). First strand cDNA synthesis was carried out using the QuantiTec Reverse Transcription Kit (Qiagen). Resulting cDNA concentrations were determined with a Qubit Fluorimeter (Invitrogen).

### Library construction and 454 sequencing

Construction of two tagged (TCTACT bud/TGTATC wt) 3’-fragment cDNA libraries from *C. vulgaris* flower tissue of the bud bloomer ‘Maria’ and its wild type flowering offspring F1 and subsequent 454 sequencing was performed by vertis Biotechnolgie AG, Freising, Germany. Quality checked and adapter trimmed sequences were obtained in fastq format sorted according to the sequence tag. Obtained fastq files were split into fasta and qual files with MIRA 3.0.5 [57] by the convert_project command. For expression analysis, sequences from plastids, endophytes, mitochondria, and for rRNA, were removed using SeqClean [58].

### Sequence annotation, read number determination and expression analysis

Sequence reads were assembled and mapped using the cDNA option of GS DeNovoAssembler (Newbler) 2.5.3 (Roche). BLAST search (blastx, NCBI nr, 1.0 E-3), mapping and annotation (default options) was performed in blast2go [27]. Three transcriptome data bases were obtained: two tag-sorted specific for one flower type each, and a common one containing both libraries (backbone). For in silico expression analysis, transcript abundances were obtained by mapping the flower type specific reads to the common backbone. Only contigs containing more than two reads were used in transcript profiling. Differentially expressed contigs were identified using the Audic Claverie algorithm [28] (p = 0.01, Bonferroni correction) with the web tool IDEG6 [29].

### qRT PCR analysis

Ten putative reference genes were chosen from the transcriptome data base (Table 3). Only genes annotated as known housekeeping genes and present as only one single contig in the data base were considered. Ct values for these genes were determined for all tissues. These data were transformed to relative quantities using the 2^-ΔCt^ formula. Stability of reference genes and optimal number of reference genes was evaluated using geNormPlus (embedded in qbasePlus, biogazelle) [59]. For subsequent analysis of gene expression patterns, five reference genes were recommended: *CvTATA binding protein*, *CvActin*, *Cv18S rRNA*, *TSa synthase*, and *CvDisease resistance protein* were identified as most stably expressed reference genes in different organs as leafs and bracts, perianth organs and sexual flower organs. Primers (Table 3) were designed using Primer3 [60] or OligoPerfect (Invitrogen). Prior to expression analysis, primer concentration was optimised, no-template-controls were run and primer efficiencies were determined by standard curves [61,62]. PCR reactions (three biological replicates in duplicates) were performed with 0.5 ng cDNA on a Stratagene MX3000P thermocycler (qPCR MxPro v4.01) using the Absolute qPCR SYBR green ROX mix (ABgene). The experimental data were normalised to the mean value of the reference genes using the 2^-ΔΔCt^ method [61]. *C. vulgaris* genotype F1 (wild type phenotype) was chosen as reference. The calculated relative quantity for each floral whorl is expressed as the ratio (fold change of arbitrary units) to the same tissue from F1 (wt). If the calculated value was <1 the negative reciprocal is given.

**Table 3:** **qRT PCR primers designed for amplification of products from 80-120 bp**

**Target sequence**	**Primer sequence**	**Product size in bp**
*CvDisease resistance protein* [contig02315]	Forward: GAAGTACAACGGAAGCACGA	106
	Reverse: CCTCTAGCAAACCGGAAAAG	
*CvTATA binding* [contig05402]	Forward: AACATCGTTGGTTCCTGTGA	101
	Reverse: CCAGGAAATAGTTCGGGTTC	
*CvTSa* [contig04235]	Forward: GTGCTCTTGGTTGGTTGTGA	89
	Reverse: ACAGGCATGGTCGTCTTTTC	
*Cv18S rRNA*, [GenBank: AF419791]	Forward: AGGGTTGAGGCAGAGAGAGA	117
	Reverse: AGAACCCCACAGAACCTCAG	
*CvActin* [contig03212]	Forward: GCATCACTAAGCACCTTCCA	111
	Reverse: CCCTCATCACGCAATTTAGA	
*CvAP3* [contig09453]	Forward: ACATCAGTCCCCCTTCTACG	88
	Reverse: CATAGTGCGAGCTCCAAAGA	
*CvPI* [contig07420]	Forward: CCCAATTTGCAGGATAGGTT	93
	Reverse: TCCCCATTACAGTTCCAACA	

## Availability of supporting data

The raw sequence reads and the result table from the in silico expression analysis have been deposited at NCBI Gene Expression Omnibus (GEO) database under the accession GSE60105. The transcriptome shotgun assembly projects have been deposited at NCBI GenBank under the GenBank accessions GBSW00000000 (backbone) and GBRS00000000 (flower type specific). The versions described in this paper are the first versions, GBSW01000000 and GBRS01000000.

## Additional files

Additional file 1:
**List of species giving BLAST Top-Hits with number of BLAST hits per assembly.**
Additional file 2:
**List of all differentially expressed contigs, coloured marked when listed in Wuest et al. 2012 [** 30 **], green - same expression pattern, orange – different expression pattern.**
Additional file 3:
**Subset of potential candidate genes with GO terms and transcript abundances.**
