# Supplementary material for: “The usual suspects”- analysis of transcriptome sequences reveals deviating B gene activity in C. vulgaris bud bloomers
Source: BMC Plant Biol. 2015 Jan 21;15:8. doi: 10.1186/s12870-014-0407-z (PMC4312453; doi:10.1186/s12870-014-0407-z)
Supplement: Additional file 1: — List of species giving BLAST Top-Hits with number of BLAST hits per assembly. [file 12870_2014_407_MOESM1_ESM.docx]

| Species | backbone | wt | bud |
| --- | --- | --- | --- |
| *Vitis vinifera* | 2083 | 1095 | 1295 |
| *Populus trichocarpa* | 850 | 438 | 536 |
| *Solanum lycopersicum* | 759 | 398 | 491 |
| *Prunus persica* | 652 | 376 | 418 |
| *Ricinus communis* | 711 | 348 | 405 |
| *Glycine max* | 416 | 244 | 286 |
| *Fragaria vesca* | 356 | 214 | 242 |
| *Cucumis sativus* | 307 | 183 | 223 |
| *Medicago truncatula* | 231 | 124 | 148 |
| *Lotus japonicus* | 138 | 97 | 97 |
| *Arabidopsis thaliana* | 103 | 79 | 74 |
| *Camellia sinensis* | 78 | 67 | 63 |
| *Zea mays* | 75 | 42 | 57 |
| *Oryza sativa* | 67 | 40 | 43 |
| *Nicotiana tabacum* | 48 | 40 | 37 |
| *Solanum tuberosum* | 41 | 33 | 35 |
| *Arabidopsis lyrata* | 49 | 30 | 27 |
| *Gossypium hirsutum* | 27 | 21 | 19 |
| *Picea sitchensis* | 23 | 19 | 16 |
| *Hevea brasiliensis* | 0 | 15 | 13 |
| *Sorghum bicolor* | 23 | 13 | 13 |
| *Actinidia deliciosa* | 18 | 0 | 0 |
| *Brachypodium distachyon* | 15 | 0 | 0 |
| *Triticum urartu* | 12 | 0 | 0 |
| *unknown* | 0 | 12 | 13 |
| *Dimocarpus longan* | 14 | 12 | 10 |
| *Camellia oleifera* | 12 | 12 | 9 |
| *Jatropha curcas* | 14 | 10 | 9 |
| *Olea europaea* | 14 | 10 | 8 |
| *Actinidia chinensis* | 14 | 9 | 8 |
| *Hordeum vulgare* | 15 | 9 | 8 |
| *Malus x* | 0 | 9 | 8 |
| *others* | 642 | 437 | 467 |
