# Supplementary material for: “The usual suspects”- analysis of transcriptome sequences reveals deviating B gene activity in C. vulgaris bud bloomers
Source: BMC Plant Biol. 2015 Jan 21;15:8. doi: 10.1186/s12870-014-0407-z (PMC4312453; doi:10.1186/s12870-014-0407-z)
Supplement: Additional file 2: — List of all differentially expressed contigs, coloured marked when listed in Wuest et al. 2012 [ 30 ], green - same expression pattern, orange – different expression pattern. [file 12870_2014_407_MOESM2_ESM.docx]

|  |  | wt | bud | wt | bud |  |  |  |
| --- | --- | --- | --- | --- | --- | --- | --- | --- |
| UNIQID | Annotation | Lib1 | Lib2 | Lib1(norm) | Lib2(norm) | In Wuest et al ? | Which mutant? | Same expression pattern? |
| >contig00147 | gdsl esterase lipase at5g33370-like | 106 | 171 | 5,5 | 10,9 | yes (at5g33370) | *ap3-1* | 3 d |
| >contig00151 | gdsl esterase lipase | 0 | 111 | 0 | 7,1 | yes (protein family) | All mutants | homolog dependent |
| >contig00207 | pr-10 type pathogenesis-related protein | 559 | 2846 | 28,9 | 180,1 | no |  |  |
| >contig00324 | osmotin-like protein | 587 | 2836 | 30,3 | 180,3 | no |  |  |
| >contig00339 | 3‘-N-debenzoyl-2‘-deoxytaxol N-benzolytranserfase | 1143 | 0 | 59,1 | 0 | no |  |  |
| >contig00356 | fructokinase-like protein | 94 | 25 | 4,9 | 1,6 | yes | *pi-1* | homolog depending |
| >contig00357 | pfkb-type carbohydrate kinase family protein | 37 | 0 | 1,9 | 0 | yes | *pi-1* | homolog depending |
| >contig00438 | 60s ribosomal protein l4-like | 233 | 99 | 12 | 6,3 | no |  |  |
| >contig00440 | 60s ribosomal protein l4 | 164 | 0 | 8,5 | 0 | no |  |  |
| >contig00523 | glycerol-3-phosphate acyltransferase 6 | 0 | 36 | 0 | 2,3 | yes | *pi-1*, *ap3-1* | 5d |
| >contig00526 | caffeic acid 3-o-methyltransferase 1 | 0 | 24 | 0 | 1,5 | no |  |  |
| >contig00603 | ribulose bisphosphate small subunit | 72 | 118 | 3,7 | 7,5 | yes | *pi-1* | 0d, 5d |
| >contig00615 | stress responsive a b barrel domain family protein | 2 | 725 | 0,1 | 46,1 | yes | *pi-1*, *ap3-3* | *pi-1,* 0d  ap3-3 no |
| >contig00650 | acyl carrier protein | 0 | 207 | 0 | 13,2 | no |  |  |
| >contig00797 | xyloglucan endotransglycosylase | 28 | 0 | 1,4 | 0 | no |  |  |
| >contig00966 | 60s ribosomal protein l6-like | 0 | 306 | 0 | 19,5 | no |  |  |
| >contig01029 | protein | 104 | 29 | 5,4 | 1,8 | yes (60S acidic ribosomal protein) | *pi-1* | 3d |
| >contig01122 | 60s ribosomal protein l10-like | 94 | 4 | 4,9 | 0,3 | no |  |  |
| >contig01149 | 60s ribosomal protein | 50 | 2 | 2,6 | 0,1 | no |  |  |
| >contig01237 | triosephosphate isomerase | 0 | 28 | 0 | 1,8 | yes | *ap3-1* | 0d, 3d, 5d |
| >contig01255 | probable fructose-bisphosphate aldolase chloroplastic-like | 0 | 33 | 0 | 2,1 | no |  |  |
| >contig01263 | 40s ribosomal protein s23-like | 31 | 0 | 1,6 | 0 | no |  |  |
| >contig01282 | 3-ketoacyl-synthase 6 | 32 | 0 | 1,7 | 0 | no |  |  |
| >contig01309 | 60s ribosomal protein l37 | 0 | 33 | 0 | 2,1 | no |  |  |
| >contig01315 | 60s ribosomal protein l29-1-like | 0 | 25 | 0 | 1,6 | no |  |  |
| >contig01332 | atp synthase subunit delta mitochondrial-like | 51 | 0 | 2,6 | 0 | no |  |  |
| >contig01367 | elongation factor 1-beta | 111 | 24 | 5,7 | 1,5 | yes | *ap3-3* | 0d, 3d, 6d |
| >contig01420 | 40s ribosomal protein s15-like | 179 | 0 | 9,3 | 0 | no |  |  |
| >contig01426 | flavonol synthase | 0 | 64 | 0 | 4,1 | no |  |  |
| >contig01427 | flavonol synthase | 270 | 68 | 13,1 | 4,3 | no |  |  |
| >contig01471 | protein | 263 | 66 | 13,6 | 4,2 |  |  |  |
| >contig01545 | 60s ribosomal protein l12-like | 7 | 60 | 0,4 | 3,8 | no |  |  |
| >contig01586 | pollen-specific protein c13-like | 4 | 72 | 0,2 | 4,6 | no |  |  |
| >contig01594 | elongation factor ef-1 gamma subunit | 10 | 67 | 0,5 | 4,3 | no |  |  |
| >contig01630 | 60s ribosomal protein l38 | 24 | 0 | 1,2 | 0 | no |  |  |
| >contig01652 | cytochrome b-c1 complex subunit 9-like | 0 | 35 | 0 | 2,2 | no |  |  |
| >contig01680 | phosphoglycerate kinase | 24 | 0 | 1,2 | 0 | yes | *pi-1*, *ap3-3* | *pi-1*, 3d; *ap3-3*, 3d |
| >contig01726 | 60s ribosomal protein l21 | 0 | 38 | 0 | 2,4 | no |  |  |
| >contig01855 | 26s proteasome non-atpase regulatory subunit rpn12a | 6 | 55 | 0,3 | 3,5 | no |  |  |
| >contig01943 | probable membrane-associated kinase regulator 1-like | 9 | 53 | 0,5 | 3,4 | no |  |  |
| >contig02039 | class 2 small heat shock protein Le-HSP 17.6 | 232 | 43 | 11,1 | 2,7 | no |  |  |
| >contig02383 | small nuclear ribonucleoprotein G | 0 | 25 | 0 | 1,6 | yes (Protein family) | All mutants | *ap3-1*, 0d, 3d, 6d |
| >contig03053 | 60s ribosomal protein l6-like | 102 | 0 | 5,3 | 0 | no |  |  |
| >contig03058 | cell wall protein | 184 | 77 | 9,5 | 4,9 | no |  |  |
| >contig03108 | light-regulated protein | 37 | 1 | 1,9 | 0,1 | no |  |  |
| >contig03137 | gdsl esterase lipase at3g48460-like | 56 | 154 | 2,9 | 9,8 | yes (at3g48460) | *ap3-3* | 3d |
| >contig03176 | udp-glucose:flavonoid 3-o-glucosyltransferase | 111 | 29 | 5,7 | 1,8 | no |  |  |
| >contig03221 | 60s ribosomal protein l35a | 0 | 21 | 0 | 1,3 | no |  |  |
| >contig03239 | 60s ribosomal protein l3-like | 188 | 0 | 9,7 | 0 | No |  |  |
| >contig03271 | 5-methyltetrahydropteroyltriglutamate-homocysteine-methyltransferase-like | 0 | 101 | 0 | 6,4 | no |  |  |
| >contig03298 | 40s ribosomal protein s4-like | 50 | 4 | 2,6 | 0,3 | no |  |  |
| >contig03302 | mlp-like protein 423-like | 68 | 3 | 3,5 | 0,2 | Yes (MLP-like protein 423) | *ap3-1* | no |
| >contig03326 | glutathione s-transferase, tau | 0 | 52 | 0 | 3,3 | yes (protein family) | *ap3-3*, *ap3-1* | ap3-3 and ap3-1 0d, 3d, 5d |
| >contig03335 | 60s ribosomal protein l3-like | 59 | 1 | 3 | 0,1 | no |  |  |
| >contig03346 | sucrose synthase | 131 | 29 | 6,8 | 1,8 | yes (protein family) | *ap3-1* | 0d, 3d |
| >contig03375 | pointed first leaf | 1 | 22 | 0,1 | 1,4 | no |  |  |
| >contig03379 | 60s ribosomal protein l32-1-like | 594 | 327 | 30,7 | 20,8 | no |  |  |
| >contig03414 | 60s ribosomal protein l2 | 56 | 0 | 2,9 | 0 | no |  |  |
| >contig03435 | protein tap1 precursor-like | 460 | 161 | 23,8 | 10,2 | Tapetum 1 | *ap3-3*, *ap3-1* | *ap3*-1 5d; *ap3-3* 0d |
| >contig03447 | cystathionine beta- chloroplastic-like | 0 | 185 | 0 | 11,8 | yes | *pi-1* | 0d |
| >contig03507 | s-adenosylmethionine synthetase | 12 | 49 | 0,6 | 3,1 | yes | *ap3-1* | 0d |
| >contig03532 | Uncharacterized protein TCM_038892 | 0 | 45 | 0 | 2,9 |  |  |  |
| >contig03543 | chloroplast chlorophyll a b binding protein | 24 | 0 | 1,2 | 0 | yes (protein family) | All mutants | *pi-1 3d;ap3-3 0d,3d* |
| >contig03578 | protein | 0 | 61 | 0 | 3,9 | to general |  |  |
| >contig03596 | 60s ribosomal protein l5-like | 0 | 188 | 0 | 11,1 | no |  |  |
| >contig03612 | gdsl esterase lipase at5g45910-like | 0 | 20 | 0 | 1,3 | no |  |  |
| >contig03617 | 60s ribosomal protein l3-like | 127 | 37 | 6,6 | 2,4 | no |  |  |
| >contig03649 | 60s ribosomal protein l22-2-like | 48 | 0 | 2,5 | 0 | no |  |  |
| >contig03661 | 60s ribosomal protein l17-2-like | 168 | 70 | 8,7 | 4,5 | no |  |  |
| >contig03671 | 40s ribosomal protein s19 | 0 | 23 | 0 | 1,5 | no |  |  |
| >contig03679 | probable protein pop3-like | 1 | 155 | 0,1 | 9,9 | no |  |  |
| >contig03702 | phenolic glucoside malonyltransferase 2-like | 0 | 33 | 0 | 2,1 | no |  |  |
| >contig03740 | aquaporin pip1-3 | 105 | 20 | 5,4 | 1,3 | no |  |  |
| >contig03770 | basic transcription factor 3 | 0 | 19 | 0 | 1,2 | no |  |  |
| >contig03790 | pinus taeda anonymous locus cl697contig1_03 genomic sequence | 185 | 1 | 9,6 | 0,1 |  |  |  |
| >contig03840 | histone h4 | 45 | 0 | 2,3 | 0 | yes | *ap3-3* | 0d, 3d, 5d |
| >contig03876 | 60s ribosomal protein l36-2-like | 345 | 411 | 17,8 | 26,1 | no |  |  |
| >contig03879 | 40s ribosomal protein s28-like | 54 | 139 | 2,8 | 8,8 | no |  |  |
| >contig03979 | 40s ribosomal protein s25-2-like | 24 | 0 | 1,2 | 0 | no |  |  |
| >contig04035 | dnaj protein homolog | 53 | 1 | 2,7 | 0,1 | yes (multiple homologs) | All mutants | homolog dependent |
| >contig04201 | ethylene-responsive transcription factor win1-like | 111 | 18 | 5,7 | 1,1 | no |  |  |
| >contig04257 | 60s ribosomal protein l37a-like | 70 | 0 | 3,6 | 0 | no |  |  |
| >contig04267 | acetolactate synthase | 182 | 64 | 9,4 | 4,1 | no |  |  |
| >contig04314 | protein | 3 | 34 | 0,2 | 2,2 |  |  |  |
| >contig04336 | 60s ribosomal protein l35-like | 0 | 23 | 0 | 1,5 | no |  |  |
| >contig04431 | 60s ribosomal protein l38 | 164 | 38 | 8,5 | 2,4 | no |  |  |
| >contig04438 | 60s ribosomal protein l13a | 0 | 37 | 0 | 2,4 | no |  |  |
| >contig04500 | ring-h2 finger protein atl48-like | 38 | 1 | 1,1 | 0,1 |  |  |  |
| >contig04519 | glutathione S-transferase, phi | 169 | 224 | 8,7 | 14,2 | yes | *ap3-1*, *ap3-3* | *ap3-1* 5d, *ap3-3* 5d |
| >contig04545 | galactinol synthase | 0 | 110 | 0 | 6,1 | no |  |  |
| >contig04604 | 40s ribosomal protein | 0 | 29 | 0 | 1,8 | no |  |  |
| >contig04872 | 70 kda peptidyl-prolyl isomerase | 1 | 22 | 0,1 | 1,4 | yes | All mutants | *ap3-3 0d,3d, 6d*, *ap3-1* 0d *pi homolog dependent* |
| >contig04910 | ethylene-responsive transcription factor rap2-3 | 27 | 0 | 1,4 | 0 | no |  |  |
| >contig05171 | 40s ribosomal protein s3a-like | 11 | 47 | 0,6 | 2,1 | no |  |  |
| >contig05190 | mitochondrial import receptor subunit tom6 homolog | 0 | 20 | 0 | 1,3 | no |  |  |
| >contig05256 | transcription factor asg4-like | 0 | 30 | 0 | 1,9 | no |  |  |
| >contig05333 | 40s ribosomal protein s26-1-like | 56 | 109 | 2,9 | 6,9 | no |  |  |
| >contig06170 | dihydrofolate reductase-like | 87 | 0 | 4,5 | 0 | no |  |  |
| >contig06238 | 40s ribosomal protein s29 | 250 | 37 | 12,9 | 2,4 | no |  |  |
| >contig06314 | dead-box atp-dependent rna helicase 5-like | 0 | 29 | 0 | 1,8 | yes (protein family) | *pi-1*, *ap3-3* | *pi-1* 0d,3d 5d, *ap3-3 0d,3d 5d* |
| >contig06385 | heat shock factor protein hsf24-like | 43 | 0 | 2,2 | 0 | no |  |  |
| >contig06667 | 40s ribosomal protein s30 | 0 | 21 | 0 | 1,3 | no |  |  |
| >contig06710 | arabinogalactan protein 20 | 29 | 0 | 1,5 | 0 | no |  |  |
| >contig06808 | 40s ribosomal protein s29 | 25 | 0 | 1,3 | 0 | no |  |  |
| >contig07583 | udp-glycosyltransferase 73c5-like isoform x1 | 116 | 35 | 5,1 | 2,2 | no |  |  |
| >contig07601 | cxe carboxylesterase | 67 | 0 | 3,5 | 0 | no |  |  |
| >contig07811 | histone h4 | 37 | 1 | 1,9 | 0,1 | no | *ap3-3* | no |
| >contig08255 | PREDICTED: uncharacterized protein LOC100841706 | 320 | 159 | 16,5 | 10,1 |  |  |  |
| >contig08472 | 40s ribosomal protein s29 | 0 | 19 | 0 | 1,2 | no |  |  |
| >contig08677 | hxxxd-type acyl-transferase-like protein | 85 | 1 | 4,4 | 0,1 | no |  |  |
| >contig09444 | 60s ribosomal protein l39 | 53 | 8 | 2,7 | 0,5 | no |  |  |
| >contig10418 | anthocyanidin 3-o-glucosyltransferase 5-like | 255 | 429 | 13,2 | 27,3 | no |  |  |
| >contig10512 | ost4a_orysj ame: full=dolichyl-diphosphooligosaccharide-protein glycosyltransferase subunit 4a | 123 | 230 | 6,4 | 14,6 | yes (protein family) | *ap3-1* | 3d, 5d |
| >contig11348 | ubiquitin ligase | 77 | 0 | 3,1 | 0 | yes | *ap3-1* | 0d |
| >contig00032 | histone 2 | 0 | 31 | 0 | 1,1 | yes, other isoform |  |  |
| >contig00062 | wound-responsive family protein | 0 | 22 | 0 | 1,4 | no |  |  |
| >contig00090 | lipid transfer protein | 3 | 67 | 0,2 | 4,3 | yes | *pi-1* | 0d, 3d, 5d |
| >contig00146 | gdsl esterase lipase at5g33370-like | 96 | 27 | 4,1 | 1,7 | yes | *ap3-1* | 0d, 5d |
| >contig00281 | dehydrin protein | 703 | 366 | 36,3 | 23,3 | no |  |  |
| >contig00322 | osmotin-like protein precursor | 182 | 56 | 9,4 | 3,6 | no |  |  |
| >contig00325 | osmotin-like protein precursor | 1259 | 299 | 65,1 | 19 | no |  |  |
| >contig00539 | auxin-repressed protein ARP | 231 | 106 | 11,9 | 6,7 | no |  |  |
| >contig00573 | histone h3 | 275 | 122 | 14,2 | 7,8 | yes (protein family) | *ap3-1*, | 5d |
| >contig00617 | stress responsive a b barrel domain family protein | 69 | 169 | 3,6 | 10,7 | yes | *pi-1*, *ap3-3* | *pi-1* 0d, not in *ap3-3* |
| >contig00694 | late embryogenesis abundant protein 1 | 192 | 62 | 9,9 | 3,9 | yes | *pi-1*, *ap3-3* | *pi-1* 0d, *ap3-*3 d |
| >contig00785 | chloroplast light-harvesting chlorophyll a b-binding protein | 381 | 140 | 19,7 | 8,9 | yes | All mutants | *pi-1* 3d,*ap3-3* 0d,3d |
| >contig00812 | mitochondrial atp synthase subunit | 438 | 228 | 22,6 | 14,5 | no |  |  |
| >contig00998 | nucleic acid binding protein | 1 | 86 | 0,1 | 5,5 | To general |  |  |
| >contig01031 | protein | 113 | 22 | 5,8 | 1,4 |  |  |  |
| >contig01100 | photosystem ii 5 kda chloroplastic-like | 4 | 51 | 0,2 | 3,2 | no |  |  |
| >contig01175 | ubiquitin-conjugating enzyme e2-17 kda | 102 | 18 | 5,3 | 1,1 | no |  |  |
| >contig01218 | metallothionein-like protein | 0 | 37 | 0 | 2,4 | no |  |  |
| >contig01225 | histone h4 like | 22 | 62 | 1,1 | 3,9 | yes | *ap3-3* | 0d, 3d, 5d |
| >contig01256 | probable fructose-bisphosphate aldolase chloroplastic-like | 47 | 2 | 2,4 | 0,1 | no |  |  |
| >contig01283 | 3-ketoacyl- synthase | 129 | 49 | 6,7 | 3,1 | no |  |  |
| >contig01316 | 60s ribosomal protein l29-1-like | 73 | 17 | 3,8 | 1,1 | no |  |  |
| >contig01368 | metallothionein-like protein | 182 | 43 | 9,4 | 2,7 | no |  |  |
| >contig01409 | iron-sulfur cluster assembly protein 1-like | 64 | 13 | 3,3 | 0,8 | no |  |  |
| >contig01421 | 40s ribosomal protein s15-like | 155 | 46 | 8 | 2,9 | no |  |  |
| >contig01472 | protein | 28 | 84 | 1,4 | 5,3 |  |  |  |
| >contig01509 | sterol desaturase-like partial | 63 | 0 | 3,3 | 0 | no |  |  |
| >contig01612 | patatin t5 | 207 | 78 | 10,7 | 4,1 | no |  |  |
| >contig01698 | polyubiquitin-like protein | 75 | 20 | 3,9 | 1,3 | no |  |  |
| >contig01719 | protein | 3 | 41 | 0,2 | 2,6 |  |  |  |
| >contig01727 | 60s ribosomal protein l21 | 141 | 49 | 7,3 | 3,1 | no |  |  |
| >contig02163 | histone h1 | 95 | 15 | 4,9 | 0,1 | no |  |  |
| >contig02313 | disease resistance-responsive (dirigent-like protein) family protein | 7 | 38 | 0,4 | 2,4 | homologs | *ap3-1* (2),  *pi-1* (1) | *ap3-1* 0d/0d 3d, *pi*1-0d,3d |
| >contig02347 | uncharacterized loc101214978 | 99 | 26 | 5,1 | 1,7 |  |  |  |
| >contig03054 | uncharacterized protein LOC100813444 | 16 | 51 | 0,8 | 3,2 |  |  |  |
| >contig03191 | lipid transfer protein | 186 | 38 | 9,6 | 2,4 | yes | *pi-1* | no |
| >contig03201 | glutathione peroxidase | 0 | 29 | 0 | 1,8 | yes (other isoform) | *pi-1* | 3d, 5d |
| >contig03248 | gaga-binding transcriptional activator | 56 | 142 | 2,9 | 9 | no |  |  |
| >contig03342 | gibberellin-regulated protein 3 | 75 | 333 | 3,9 | 21,2 | yes (protein family) | *pi-1*(2),  *ap3-3* (1) | *pi-1* 0d, 3d |
| >contig03361 | awpm-19-like family protein | 205 | 341 | 10,6 | 21,7 | no |  |  |
| >contig03444 | probable glycerol-3-phosphate acyltransferase 8-like | 18 | 63 | 0,9 | 4 | yes (other isoform) | All mutants | Time dependent |
| >contig03511 | asr2 protein | 17 | 55 | 0,9 | 3,5 | no |  |  |
| >contig03588 | low temprature induced-like protein | 0 | 54 | 0 | 3,4 | no |  |  |
| >contig03591 | chitinase-like protein 2 | 7 | 47 | 0,4 | 2,1 | yes (protein family) | *pi-1* | 0d, 3d, 5d |
| >contig03663 | protein | 32 | 0 | 1,7 | 0 |  |  |  |
| >contig03801 | chalcone synthase | 23 | 65 | 1,2 | 4,1 | yes (protein family) | *ap3-1* | no |
| >contig03805 | atp-dependent clp protease proteolytic subunit-related protein chloroplastic-like | 28 | 76 | 1,4 | 4,8 | no |  |  |
| >contig03997 | uncharacterized loc101216438 | 46 | 6 | 2,4 | 0,4 |  |  |  |
| >contig04185 | kda sulfur-rich protein | 12 | 71 | 0,6 | 4,5 | no |  |  |
| >contig04375 | basic blue protein | 62 | 0 | 3,2 | 0 | yes | *pi-1* | 3d |
| >contig04747 | protein ethylene insensitive 3-like | 48 | 0 | 2,5 | 0 | no |  |  |
| >contig04851 | pinus taeda anonymous locus 2_4562_01 genomic sequence | 2 | 28 | 0,1 | 1,8 |  |  |  |
| >contig04874 | casp-like protein vit_07s0104g01350-like | 10 | 396 | 0,5 | 25,2 | no |  |  |
| >contig04908 | stem-specific protein tsjt1-like | 398 | 216 | 20,6 | 13,7 | no |  |  |
| >contig05034 | heat shock protein 70 | 178 | 71 | 9,2 | 4,5 | yes | *ap3-1* | 0d, 3d |
| >contig05162 | Uncharacterized protein TCM_037897 | 56 | 119 | 2,9 | 7,6 |  |  |  |
| >contig05488 | udp-glycosyltransferase 73c5-like | 25 | 61 | 1,3 | 3,9 | no |  |  |
| >contig05824 | defensin | 0 | 39 | 0 | 2,5 | Yes(different homologs) | All mutants | yes depending on homolog |
| >contig05857 | flavonoid 3 -hydroxylase | 0 | 19 | 0 | 1,2 | no |  |  |
| >contig05943 | dihydrofolate reductase-like | 0 | 20 | 0 | 1,3 | no |  |  |
| >contig06023 | glutaredoxin-like protein | 54 | 1 | 2,8 | 0,1 | Different homologs | All mutants | yes depending on homolog |
| >contig06053 | PREDICTED: uncharacterized protein LOC101505687 | 0 | 76 | 0 | 4,8 |  |  |  |
| >contig06193 | auxin-repressed protein | 38 | 3 | 1,1 | 0,2 | no |  |  |
| >contig06356 | 14-3-3 protein | 51 | 1 | 2,6 | 0,1 | no |  |  |
| >contig06404 | alpha- galactosidase | 0 | 43 | 0 | 2,7 | no |  |  |
| >contig07071 | protein | 1 | 23 | 0,1 | 1,5 |  |  |  |
| >contig07181 | calcium-dependent protein kinase 4-like | 0 | 31 | 0 | 1,1 | yes | *ap3-3* | no |
| >contig07341 | udp-glycosyltransferase 79b3-like | 5 | 60 | 0,3 | 3,8 | no |  |  |
| >contig07420 | mads-domain transcription partia (PI)l | 48 | 6 | 2,5 | 0,4 | yes | *pi-1*, *ap3-3* | *pi-1* and *ap3-3* 0d, 3d, 5d |
| >contig07413 | cysteine proteinase | 108 | 0 | 5,6 | 0 | yes( protein family) | All mutants | Depending on homolog |
| >contig07453 | uncharacterized loc101214579 | 45 | 1 | 2,3 | 0,1 |  |  |  |
| >contig07866 | mitochondrial import receptor subunit tom6 homolog | 0 | 265 | 0 | 16,9 | no |  |  |
| >contig08053 | protein srg1-like | 1 | 21 | 0,1 | 1,3 | no |  |  |
| >contig08253 | e2f transcription factor-like e2fe-like | 0 | 19 | 0 | 1,2 | no |  |  |
| >contig08852 | early dehydration inducible protein | 0 | 23 | 0 | 1,5 | no |  |  |
| >contig09796 | hyaluronan mrna binding family isoform 1 | 0 | 27 | 0 | 1,7 | no |  |  |
| >contig10308 | structural maintenance of chromosomes 1 | 55 | 0 | 2,8 | 0 |  |  |  |
| >contig10710 | desiccation protectant protein lea14-like protein | 85 | 161 | 4,4 | 10,2 | no |  |  |
| >contig11378 | cysteine proteinase inhibitor | 0 | 18 | 0 | 1,1 | no |  |  |
| >contig11379 | kinesin-like protein kin12b-like | 0 | 24 | 0 | 1,5 | Yes (protein family) | *pi-1* | 3d |
| >contig11731 | uncharacterized loc101211888 | 8 | 35 | 0,4 | 2,2 |  |  |  |
