# Supplementary material for: “The usual suspects”- analysis of transcriptome sequences reveals deviating B gene activity in C. vulgaris bud bloomers
Source: BMC Plant Biol. 2015 Jan 21;15:8. doi: 10.1186/s12870-014-0407-z (PMC4312453; doi:10.1186/s12870-014-0407-z)
Supplement: Additional file 3: — Subset of potential candidate genes with GO terms and transcript abundances. [file 12870_2014_407_MOESM3_ESM.docx]

| Contig | GO term | Annotation | Function | Raw read number bud | Raw read number wt | Normalised read number bud | Normalised read number wt |
| --- | --- | --- | --- | --- | --- | --- | --- |
| 04035 | flower development | DNAj protein | co-chaperon of HSP 70 [63, 64] | 73 | 17 | 3,80 | 1,10 |
| 00523 | flower development | Glycerol-3-Phosphate Acyltransferase 6 | cutin biosynthesis in sepals and petals [65], tapetum development [66] | 130 | 49 | 6,70 | 3,10 |
| 01855 | flower development | 26S proteasom non atpase regulatory subunit rpu 12a protein | proteasome function, cell enlargement, cell proliferation [67] | 41 | 1 | 2,10 | 0,10 |
| 04375 | flower development/ Floral whorl development | Basic blue protein | photosynthesis [68, 69] | 28 | 84 | 1,40 | 5,30 |
| 01282 | flower development/ Floral whorl development | 3-ketoacyl-synthase 6 | long-chain fatty acid condensing, cuticular wax [70], pollen fertility | 182 | 64 | 9,40 | 4,10 |
| 03248 | sequence specific DNA binding transcription factor activity | GAGA binding transcriptional activator | response to ethylene, developmental process, ubiquitously expressed [71] | 27 | 68 | 1,40 | 4,30 |
| 04910 | sequence specific DNA binding transcription factor activity | Ethylene responsive transcription factor *RAP2-3* | ethylene- mediated plant defence responses [72] | 113 | 38 | 5,80 | 2,40 |
| 07420 | sequence specific DNA binding transcription factor activity | MADS box transcription factor *PI* | stamen and petal identity [10] | 0 | 20 | 0,00 | 1,30 |
| 08253 | sequence specific DNA binding transcription factor activity | E2FE like transcription factor | control of cell  proliferation [73],  meiosis pollen | 5 | 60 | 0,30 | 3,80 |

Contig sequences can be found in GenBank under the GenBank accession number GBSW00000000. The raw sequence reads and the result table from the in silico expression analysis have been deposited at NCBI Gene Expression Omnibus (GEO) database under the accession number GSE60105.

63. Walsh P, Bursac D, Law YC, Cyr D, Lithgow T. The J-protein family: modulating protein assembly, disassembly and translocation. EMBO Rep. 2004;5(6):567–71.

64. Liberek K, Lewandowska A, Zietkiewicz S. Chaperones in control of protein disaggregation. EMBO J. 2008;27(2):328–35.

65. Li-Beisson Y, Pollard M, Sauveplane V, Pinot F, Ohlrogge J, Beisson F. Nanoridges that characterize the surface morphology of flowers require the synthesis of cutin polyester. Proc Natl Acad Sci. 2009;106(51):22008–13.

66. Li X-C, Zhu J, Yang J, Zhang G-R, Xing W-F, Zhang S, Yang Z-N: Glycerol-3-Phosphate Acyltransferase 6 (GPAT6) is important for tapetum development in Arabidopsis and plays multiple roles in plant fertility. Molecular Plant 2011;5(1): 131-142

67. Kurepa J, Wang S, Li Y, Zaitlin D, Pierce AJ, Smalle JA. Loss of 26S proteasome function leads to increased cell size and decreased cell number in *Arabidopsis* shoot organs. Plant Physiol. 2009;150(1):178–89.

68. Baker EN. Copper proteins with type I sides. In: King RB, editor. Encyclopedia of Inorganic Chemistry. Chichester, UK: Wiley Intersciences; 1994. p. 883–923.

69. Sykes AG. Active-site properties of the blue copper proteins. In: Sykes AG, editor. *Advances in Inorganic Chemistry.* Volume 36: Xiii 503p. San Diego, California, USA; London, England, UK: Academic; 1991. p. 377–408.

70. Millar AA, Clemens S, Zachgo S, Giblin EM, Taylor DC, Kunst L. *CUT1*, an *Arabidopsis* gene required for cuticular wax biosynthesis and pollen fertility, encodes a very-long-chain fatty acid condensing enzyme. Plant Cell. 1999;11(5):825–38.

71. Monfared MM, Simon MK, Meister RJ, Roig-Villanova I, Kooiker M, Colombo L, et al. Overlapping and antagonistic activities of *BASIC PENTACYSTEINE* genes affect a range of developmental processes in *Arabidopsis*. Plant J. 2011;66(6):1020–31.

72. Li H-Y, Xiao S, Chye M-L. Ethylene- and pathogen-inducible *Arabidopsis* acyl-CoA-binding protein 4 interacts with an ethylene-responsive element binding protein. J Exp Bot. 2008;59(14):3997–4006.

73. Kosugi S, Ohashi Y: E2Ls, E2F-like repressors of Arabidopsis that bind to E2F-sites in a monomeric form. Journal of Biological Chemistry 2002:77(19):16553-8
